# Efficacy of Phenotype-vs. Genotype-Guided Therapy Based on Clarithromycin Resistance for *Helicobacter pylori* Infection in Children

**DOI:** 10.3389/fped.2022.854519

**Published:** 2022-03-29

**Authors:** Yan Feng, Wenhui Hu, Yuhuan Wang, Junping Lu, Ye Zhang, Zifei Tang, Shijian Miao, Ying Zhou, Ying Huang

**Affiliations:** Department of Gastroenterology, Children's Hospital of Fudan University, Shanghai, China

**Keywords:** *Helicobacter pylori*, tailored therapy, clarithromycin resistance, phenotype, genotype

## Abstract

**Background:**

Clarithromycin resistance reduces the eradication rate of *Helicobacter pylori (H. pylori)*. Cultures with susceptibility testing and molecular determination of genotypes are recommended to guide-tailored therapy.

**Methods:**

We retrospectively enrolled patients aged 6 and 18 years with *H. pylori* infection, who underwent an endoscopy and agreed to undergo both culture and genetic testing for clarithromycin resistance. Patients receiving tailored therapy based on traditional culture results (phenotype-guided therapy) or genetic testing results (genotype-guided therapy) were included in the study. ^13^C-urea breath test was used to evaluate the success of eradication at least 4 weeks after the completion of treatment. We aimed to determine whether the eradication rate of phenotype- or genotype-guided therapy based on clarithromycin resistance is greater than 90% in children.

**Results:**

Between September 2017 and October 2020, 226 eligible patients were enrolled. There were 71 with clarithromycin-sensitive strains in the phenotype-guided therapy group and 87 without 23S rRNA point mutations (A2142G, A2142C, and A2143G) in the genotype-guided therapy group. Eradication rates were 70.4% (50/71, [95% CI: 58.4–80.7%] for phenotype-guided therapy and 92.0% (80/87, [95% CI: 84.1–96.7%]) for genotype-guided therapy (*P* < 0.01). The incidence of side effects was 4.2% (3/71) and 10.3% (9/87), with no major differences between these two groups (*P* = 0.15), respectively. The compliance rate was also similar (97.2 vs. 95.4%, *P* = 0.87).

**Conclusion:**

Tailored therapy according to genetic testing results achieved eradication rates of 92% and was superior to tailored therapy guided by traditional culture results.

## Introduction

*Helicobacter pylori* (*H. pylori*) adheres to the gastric epithelium and colonizes in the mucus layer. The relationship between *H. pylori* infection and constant chronic gastritis, peptic ulcers, and gastric cancer has been established (1). At present, more than 30% of children have been infected with *H. pylori* around the world (2). Both in developing and developed countries, *H. pylori* infection has been a serious public health problem.

In the 1990s, the first-line treatment for *H. pylori* infection was triple therapy with a 90% eradication rate and few adverse reactions. Sequential therapy previously widely used in Italy showed a higher eradication rate of 95% (3). However, due to antibiotic resistance, especially for clarithromycin (CLA), the cure rate of triple therapy and sequential therapy has reduced to approximately 60–80% (4), which is not currently acceptable in clinical practice. For children, limited numbers of antibiotics can be used for rescue therapy, so it is essential to raise the eradication rate of first-line treatment. In general, treatment failure will increase healthcare utilization and the risk of secondary antibiotic resistance. Antibiotic susceptibility tests, therefore, seem to be the logical and correct approach to follow before therapy. Indeed, the latest guidelines of ESPGHAN/NASPGHAN recommend that eradication treatment should be tailored according to the results of antimicrobial susceptibility testing (5).

There are 2 main types of methodology to assess susceptibility. Traditional culture-based antimicrobial susceptibility tests are time-consuming, complicated, and relatively costly, and the positive culture rate is between 75 and 90%. Bacterial susceptibility to CLA can also be assessed by genetic testing, which offers rapid and highly accurate results and allows for culture-free assessment. There are mainly three-point mutations of A2142C, A2142G, and A2143G associated with CLA resistance in the peptidyl transferase loop of the 23S rRNA gene, resulting in a conformational change and reducing drug binding (6). Studies have shown that mutation at these two sites can predict resistance in more than 90% of strains (7, 8).

We used the terms “*Helicobacter*,” “tailored therapy,” “genotype guide,” “culture guide,” and “susceptibility test” to search in PubMed for papers published between January 2000 and December 2021 in English. Thirteen studies investigating the effectiveness of culture-based tailored therapy in children are shown in Table 1. The eradication rate was over 90% in five studies (9, 11, 17, 20, 21) but ranged from 70 to 90% in the other eight studies (10, 12–16, 18, 19). For the tailored therapy based on molecular tests with gastric biopsy, there is a paucity of data for children, though the eradication rate in most adult studies was over 90% (22–25). Therefore, this study was conducted to confirm whether the eradication rate of phenotype-guided therapy or genotype-guided therapy based on CLA resistance is greater than 90% in Chinese children. Side effects and compliance for the two regimens were also assessed.

**Table 1:** Researches of tailored therapy in children.

**References**	**Design**	**Method**	**CLA resistance rate**	**Tailored eradication rate**
Miyata et al. (9)	retro	AST	71.1% (32/45)	14 days triple therapy 97.8% (44/45)
Kori et al. (10)	retro	AST	24.8% (244/984)	7–14 days triple therapy 79.8% (568/712)
Silva et al. (11)	pro	AST, real-time PCR	AST 23.3% (7/30) PCR 37.3% (19/51)	14 days triple therapy 97.8% (44/45)
Butenko et al. (12)	retro	AST	23.4% (25/107)	7–14 days triple therapy 85.9% (61/71)
Kotilea et al. (13)	pro	AST	6.9% (10/145)	10 days PAC 76.9% (60/78) 10 days sequential therapy 92.3% (36/39)
Montes et al. (14)	retro	AST	34.7% (25/72)	PAC 80% (20/25)
Bontems et al. (15)	pro	AST	16% (24/150)	7 days triple therapy 80.4% (37/46) 10 days sequential therapy 93.5% (43/46)
Vécsei et al. (16)	retro	AST	34.1% (14/41)	7 days PAC 69.2% (18/26)
		Fecal PCR	3.6% (2/55)	7 days PAC 78.8% (41/52)
Arenz et al. (17)	pro	AST	8.6% (5/58)	7 days PAC 92.5% (49/53)
Lopes et al. (18)	retro	AST	35.1% (33/94)	10 days triple therapy 74.7% (59/79)
Faber et al. (19)	retro	AST	15.2% (16/105)	PAC 75% (33/44)
Kato et al. (20)	retro	AST	34.7% (25/72)	7–14 days PAC 91.7% (44/48)
Street et al. (21)	pro	AST	15.9% (10/63)	8 days triple therapy 98.4% (62/63)

*retro, retrospective study; pro, prospective study; AST, antibiotic susceptibility test; CLA, clarithromycin; PAC, proton-pump inhibitor, amoxicillin and clarithromycin*.

## Materials and Methods

### Study Design and Patients

This is a retrospective study, approved by the ethics committee of the Children's Hospital of Fudan University [(2017) 186] and conducted in the outpatient department. Informed consent for enrollment and samples collection was obtained from parents.

Patients aged between 6 and 18 years with *H. pylori* infection were referred for endoscopy due to gastrointestinal symptoms, and who agreed to undergo both culture and genetic testing for CLA resistance were eligible for enrollment. Basic data of demographic, clinical, and endoscopic were prospectively collected from September 2017 to October 2020. Patients receiving tailored therapy based on traditional culture (phenotype-guided therapy) or genetic testing (genotype-guided therapy) results were ultimately included. Positively in both the rapid urease test (RUT) and histology or culture positive can be diagnosed with active *H. pylori* infection according to the latest ESPGHAN/NASPGHAN guidelines (5). Meeting any one of the criteria were excluded: use of antibiotics or bismuth agents within 4 weeks or proton pump inhibitors within 2 weeks before the examination; previous eradication therapy; allergies or contraindications to the therapeutic drugs in this study; or complications with other chronic or serious diseases.

Eligible patients were treated with 14 days of triple therapy or sequential therapy and prescribed the following drugs: omeprazole [1.0 mg/(kg·d), maximum 40 mg, once or two times a day], amoxicillin [50 mg/(kg·d), maximum 2 g, three times or four times a day], CLA [20 mg/(kg·d), maximum 1 g, two times daily], and metronidazole [20 mg/(kg·d), maximum 1 g, two times or three times a day]. Omeprazole was given 30 min before meals, and antibiotics were 30 min after meals.

Before treatment, the essential of full compliance was informed, potential adverse events also were warned and asked to record, like nausea, vomiting, diarrhea, abdominal discomfort, dizziness, headache, bitter taste, darkened stool/tongue, and rash. After the completion of the eradication therapy, side effect to treatment was assessed using a specific questionnaire during a structured clinical interview, which was classified to “mild” (can be tolerated and transient), “moderate” (partially affecting daily activities and discomfort felling), “severe” (considerable affecting daily activities), and “serious” (loss of function or life-threatening). To confirm patients' compliance, we counted the remaining pills. Patients who took ≤ 80% of the medications were considered poor compliance. The success of eradication was monitored by the ^13^C-urea breath test at least 4 weeks after treatment.

### Determination of Phenotypic and Genotypic Resistance

All patients agreed to receive gastroscopy and biopsy. Besides RUT and histology examination, another two antral specimens were taken for *H. pylori* culture and genotyping of 23S rRNA. The gastric biopsy was cultured and further used for antibiotic susceptibility testing with the epsilon-meter (E) test. Strains were considered resistant to CLA when the minimum inhibitory concentration (MIC) > 0.5 mg/l according to the European Committee on Antimicrobial Susceptibility Testing.

Deoxyribonucleic acid from gastric biopsies was extracted with the TIANamp Micro DNA Kit (DP316, Beijing, China). PCR-based amplification of the 23S rRNA was performed to detect CLA-resistant mutations (A2142G, A2142C, and A2143G). Primers as followed (Jieli Bio): forward, 1,820–1,839 (5′-CCCAGCGATGTGGTCCAG-3′) and reverse, 2,244–2,225 (5′-CTCCATAAGAGCCAAAGCCC-3′) (26). Reaction in 25-μl mixture contained 1 μl of template DNA (<1 ug), 1 μl of each primer (10 μM), 22 μl of 1.1 × T3 super PCR mix (Tsingke, TSE030) and conducted using the following program: 1 cycle at 98°C for 3 min, 35 cycles at 98°C for 15 s, 65°C for 15 s, and 72°C for 30 s and final 1 cycle at 72°C for 7 min. The 425-bp PCR products were sent to a biotechnology company for sequencing (ABI 3730XL, Jieli Bio). Finally, Chromas 2 software was used to analyze mutations at positions 2,142 and 2,143.

### Statistical Analysis

Data analysis used the software of SPSS 25.0. Classification variables were described by the frequency and percentage, continuous variables were presented by the mean and SD. All the patients who accepted at least one dose of the treatment with available follow-up data were included in the full analysis set (FAS). The chi-square test or Fisher test was applied to compare the eradication rate of each tailored therapy regimen in the analysis of FAS and *P* < 0.05 proved a significant difference.

## Results

### Characteristics of the Subject

**Figure 1:**
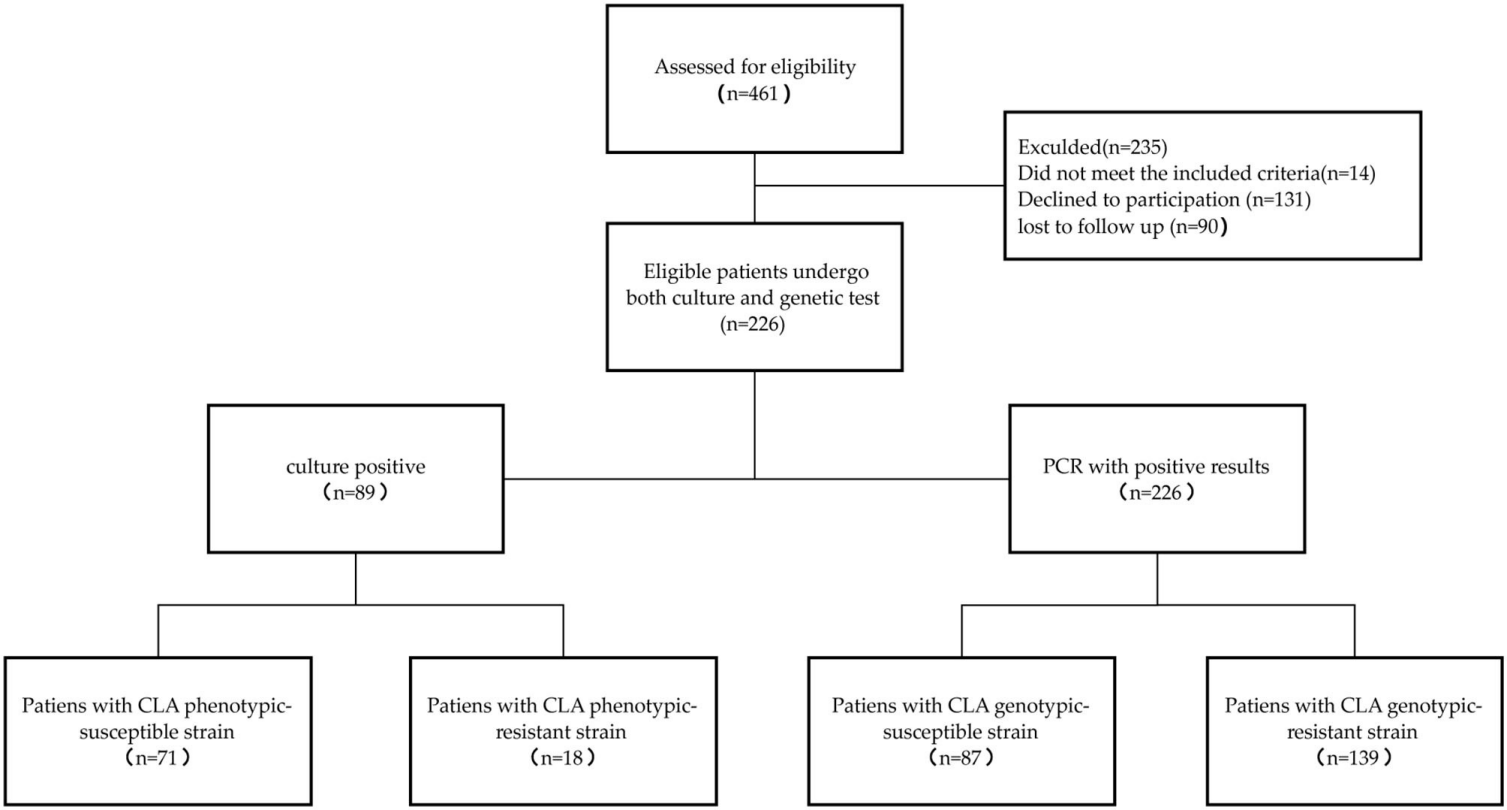
Flow chart of participant selection.

From September 2017 to October 2020, 226 eligible patients with follow-up data underwent both biopsy culture and molecular testing. The flowchart of the participant selection process is shown in Figure 1. A total of 71 patients with CLA-susceptible strains were allocated to the phenotype-guided therapy group and 87 patients without point mutations to the genotype-guided therapy group. Table 2 presents the clinical characteristics of the two groups with no significant differences in the baseline.

**Table 2:** Basic characteristics and antibiotic resistance rate of the subjects.

	**Phenotype-guided therapy group** **(*n* = 71)**	**Genotype-guided therapy group** **(*n* = 87)**	***P***
**Age**			
Mean ± SD (years)	10.2 ± 2.5	9.7 ± 2.6	0.23
**Sex**			
Male	67.6% (48/71)	66.7% (58/87)	0.90
Female	32.4% (23/71)	33.3% (29/87)	
**Chief compliant**			
Abdominal discomfort	77.5% (55/71)	79.3% (69/87)	0.78
Nausea or vomit	19.7% (14/71)	11.5% (10/87)	0.15
Other symptoms	12.7% (9/71)	14.9% (13/87)	0.68
**Endoscopic findings**			
PUD	18.3% (13/71)	12.6% (11/87)	0.34
NUD	81.7% (58/71)	87.4% (76/87)	
**Phenotype resistance**			
AMO	2.8% (2/71)	0	1.00
MET	31.0% (22/71)	43.3% (13/30)	0.23

*PUD, peptic ulcer disease; NUD, non-ulcer disease; AMO, amoxicillin; MET, metronidazole*.

### Eradication Rate

**Figure 2:**
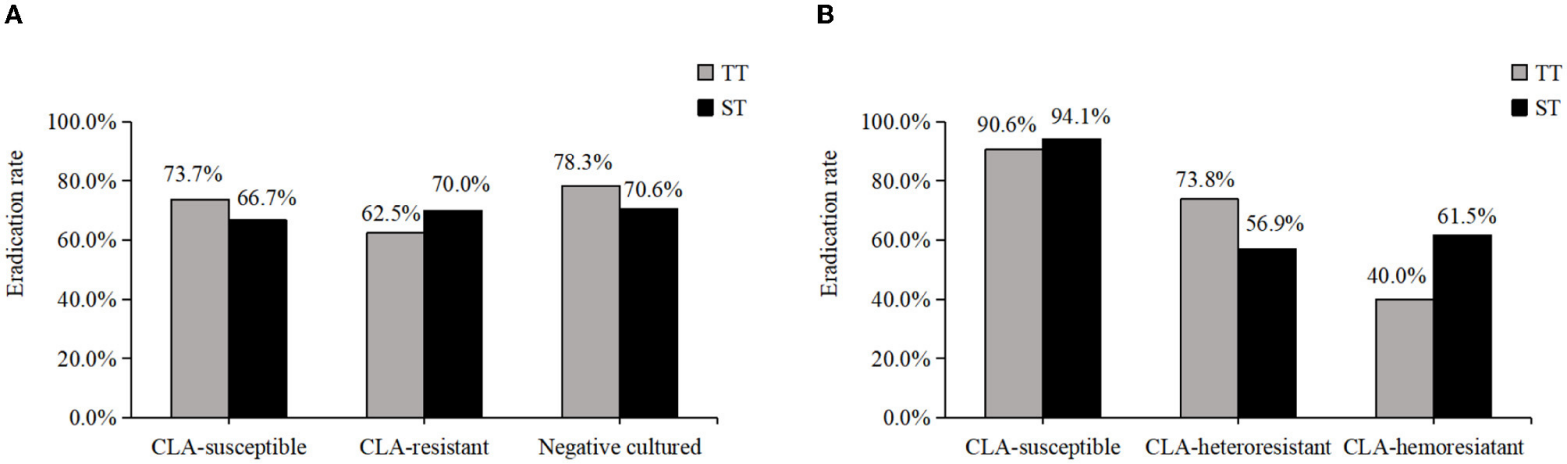
The eradication rate of *H. pylori* based on phenotype and genotype. **(A)** The eradication rate of CLA-susceptible, CLA-resistant and cultured negative strains in phenotype. **(B)** The eradication rate of CLA-susceptible, CLA-heteroresistant and CLA-hemoresistant strains in genotype.

Treatment success was achieved in 70.4% (50/71, [95% CI: 58.4–80.7%]) of patients under phenotype-guided therapy and in 92.0% (80/87, [95% CI: 84.1–96.7%]) of patients under genotype-guided therapy. The cure rate was significantly higher in the latter group. Among the phenotype-guided therapy group, 38 patients were prescribed triple therapy (2 weeks, omeprazole, amoxicillin, and CLA), and 33 were prescribed sequential therapy (first week, omeprazole plus amoxicillin; second week, omeprazole plus CLA and metronidazole). The eradication rates were 73.7% (28/38, [95% CI: 56.9–86.6%]) and 66.7% (22/33, [95% CI: 48.2–82.0%]), respectively. Of 87 patients in the genotype-guided group, 53 received triple therapy with an eradication rate of 90.6% (48/53, [95% CI: 79.3–96.9%]); the eradication rate was 94.1% (32/34, [95% CI: 80.3–99.3%]) with the application of sequential therapy. Triple therapy achieved a similar eradication rate as sequential therapy when using traditional culture-based or molecular-based tests. The eradication rate of *H. pylori* based on phenotype and genotype is shown in Figure 2.

### Antimicrobial Resistance

**Figure 3:**
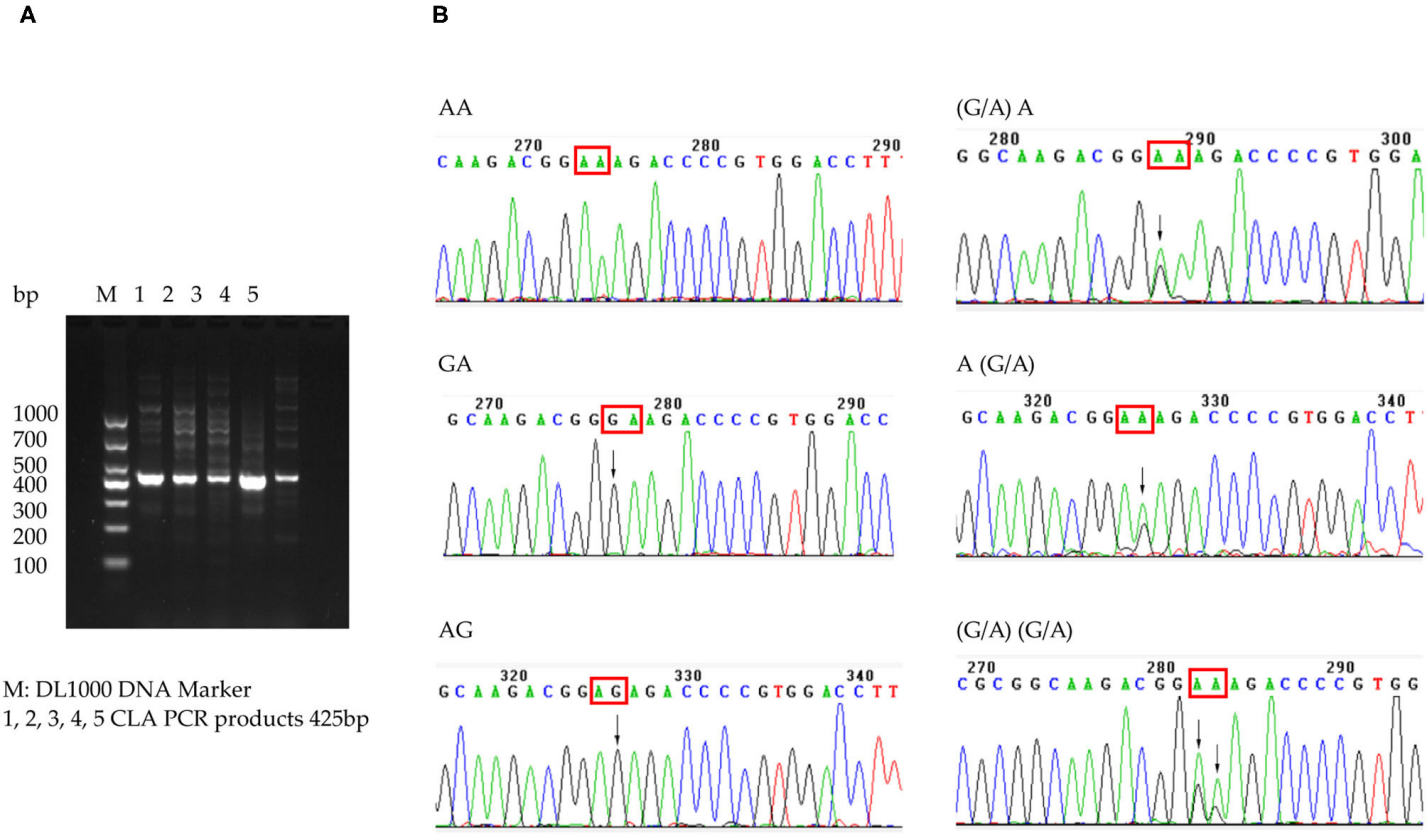
CLA 23S rRNA detected in patients. **(A)** Electrophoretogram of 23S rRNA-specific PCR products. **(B)** Sequence of CLA 23S rRNA 2142 and 2143 point mutation.

Among the 226 patients, the *H. pylori* strains of 39.4% (89/226) were successfully cultured and tested for antimicrobial susceptibility. Primary resistance rates were 20.2% (18/89) for CLA, 38.2% (34/89) for metronidazole, and 2.2% (2/89) for amoxicillin. The dual resistance rate was 13.5% (12/89) for CLA and metronidazole and 2.2% (2/89) for amoxicillin and metronidazole. A higher CLA resistance rate, at 61.5% (139/226), was detected among the 226 patients with 23S rRNA genotype results. Mutation subtypes included A2143G in 133 patients and A2142G in 22; 16 patients showed both A2143G and A2142G. In addition to heterozygotes, wild-type plus A2142G or A2143G was found, which indicated the existence of heteroresistance. The A2142C mutation was not found (Figure 3). However, phenotype and genotype not always be concordant. In total, 71 strains were CLA-susceptible in phenotype, but 45 of them detected A212G or A2143G mutation, indicating resistance in genotype. And 18 strains with CLA-resistant phenotype, but 4 strains without point mutation in 23S rRNA. The outcomes of phenotype and genotype were presented in Table 3.

**Table 3:** The results of phenotype tested by E-test and genotype detected by PCR sequencing of *H. pylori* for clarithromycin.

**Genotype**	**Phenotype**			**Total**
	**CLA-susceptible**	**CLA-resistant**	**Negative cultured**
CLA-susceptible	26	4	57	87
Heteroresistant	26	7	60	93
Hemoresistant	19	7	20	46
Total	71	18	137	226

### Side Effects and Compliance

The incidence of side effects was 4.2% (3/71) and 10.3% (9/87) following phenotype-guided therapy and genotype-guided therapy, respectively, with no major differences between the groups (shown in Table 4). All the adverse reactions were mild and disappeared spontaneously after treatment was ceased. The compliance rate was 97.2% (69/71) in the phenotype-guided therapy group and 95.4% (83/87) in the genotype-guided therapy group, similar between them (*P* = 0.87).

**Table 4:** The eradication rate, adverse reaction, and compliance of phenotype- and genotype-guided therapy group.

	**Phenotype-guided therapy group** **(*n* = 71)**	**Genotype-guided therapy group** **(*n* = 87)**	***P***
Total eradication rate	70.4% (50/71)	92.0% (80/87)	<0.01^**^
Subgroup eradication rate			
Standard triple therapy	73.7% (28/38)	90.6% (48/53)	0.03^*^
Sequential therapy	66.7% (22/33)	94.1% (32/34)	0.01^*^
Adverse events rate	4.2% (3/71)	10.3% (9/87)	0.15
Rash	66.7% (2/3)	55.6% (5/9)	0.62
Nausea or vomit	0	11.1% (1/9)	1.00
Diarrhea	33.3% (1/3)	33.3% (3/9)	0.76
Compliance	97.2% (69/71)	95.4% (83/87)	0.87

* *P < 0.05 and*

** *P < 0.01*.

## Discussion

An ideal first-line regimen is defined as highly effective with a treatment success rate above 90%, not complex with fewer drugs and with few adverse events. Our previous studies reported that 14 days of triple therapy, sequential therapy, concomitant therapy, and bismuth-based quadruple therapy achieved the eradication rate of 74.1, 69.5, 84.6, and 89.8% as first-line therapy in Chinese *H. pylori*-infected children (27), under 90%. According to ESPGHAN/NASPGHAN guidelines, antibiotics susceptibility testing should conduct in initial therapy to reach the 90% eradication rate. As CLA is the leading cause of treatment failure (28), we designed this study to assess the efficacy of phenotype-guided therapy and genotype-guided therapy based on CLA resistance in Chinese children as first-line therapy.

The success rate of phenotype-guided tailored therapy in our study was 70.4% and did not achieve the target rate of > 90%. Our results are similar to those of a respective study published by Kori et al. (10) which revealed an eradication rate of 79.8% after susceptibility-based treatment. A study in Belgium and one in Spain reported eradication rates of 72 and 78.7% in children with culture results (13, 14). There are several explanations for our low eradication rate with culture-based therapy. First, we ignored the resistance of amoxicillin and metronidazole. In the phenotype-guided therapy group, 2 patients were resistant to amoxicillin and failed in treatment. In total, 9 patients were resistant to metronidazole in the subgroup of sequential therapy with the eradication rate of 44.4% (4/9) and 24 were metronidazole sensitive, only achieved the eradication rate of 75.0% (18/24), which suggested that the resistance of amoxicillin and metronidazole has limited influence on the low eradication rate of the phenotypic group. Second, the susceptibility test *in vitro* might not reflect the actual level of antibiotics in the gastric lumen, in which there is a possible pH influence on antimicrobial activity (29). In addition, heteroresistance (coexistence of resistant and susceptible strains for the same antimicrobial agent in the patient) can lead to underestimation of antimicrobial resistance (30). Third, antibiotic resistance acquired during treatment may also be an important cause of treatment failure (31). In addition to antibiotic resistance, other factors may influence treatment efficacy, such as CYP2C19 genotype, bacterial genotype, and poor compliance (13, 32). All the patients in our study were educated about adverse events and the importance of adherence before initiating the therapy, and the compliance rates were > 95% in the two groups.

Our results showed that genotype-guided therapy acquired a higher success rate of 92.0% than phenotype-guided therapy. Molecular testing is a promising technology that is time-saving and culture-free and allows for the identification of mixed resistance. Clinicians can easily acquire gastric specimens through endoscopic biopsies. In the genotype-guided therapy group, only 30 patients cultured positive and obtained the susceptibility of antibiotics. PCR-based approaches can only detect macrolide or quinolone resistance, and they cannot be used for other antibiotics, such as amoxicillin, metronidazole, and tetracycline. However, amoxicillin always has a low-phenotypic resistance rate. As for metronidazole, Tankovic et al. (33) showed that metronidazole resistance is much more heterogeneous and has a much wider range of MIC, mainly related to various mutations in the *rdx* gene. Moreover, *in vitro* resistance cannot always predict treatment failure *in vivo* (19), and extending the duration or increasing the dose of metronidazole can partially overcome resistance to this drug (34). Genotype-guided therapy based on CLA resistance also can be recommended. Both genotype-guided triple therapy and sequential therapy exhibited efficacy above 90%. However, sequential therapy exposes the patient to 3 different antibiotics and need to be changed during treatment. Thus, genotype-guided triple therapy is preferred due to its relative simplicity.

In this study, 23S rRNA point mutations were detected in 61.5% (139/226) of *H. pylori*-infected patients, and 41.2% (93/226) were heteroresistance. According to the genotype of 23S rRNA, the wild type had the highest eradication rate, followed by heteroresistance, and hemoresistance was lowest. Although genetic testing is more sensitive and convenient, whether we highlight the resistance of CLA, more research need to confirm. Traditional cultures with susceptibility testing have the advantage of obtaining resistance to several antibiotics simultaneously. Phenotypic resistance to CLA was 20.2%, which was consistent with a preliminary study but showed a relative discordance with genotypic (PCR-based) determination of CLA resistance due to the detection of heteroresistance. And in our study, the eradication rate of CLA-susceptible strains in phenotype was only slightly higher than that of CLA-resistant. Phenotypic testing may underestimate the resistance rate.

There are several limitations to our study. First, it was a single-center, retrospective study with a limited sample size. We evaluated the correlation between outcome and antibiotic resistance retrospectively, but data about adverse events and compliance were collected prospectively. Second, only one antrum biopsy was obtained for *H. pylori* culture, which may be related to the low positive rate of culture, even underestimate antibiotic resistance, as mixed infections with multiple strains are likely to be missed. Third, we only detected mutations of 23S rRNA at positions 2142 and 2143. Other mutations or efflux pumps may also cause CLA resistance, but these two nucleotide positions are sufficient to predict more than 90% of cases of CLA resistance. Although the above limitations should be considered, this study takes the lead in evaluating the efficacy of phenotype-guided therapy and genotype-guided therapy in Chinese children.

## Conclusion

In conclusion, our study showed an association between both tailored triple therapy and sequential therapy according to the CLA-resistant mutations with a high eradication rate exceeding 90% for first-line treatment, with greater efficacy than tailored therapy based on the traditional culture-based test. Further multicenter studies with large sample sizes are needed to confirm the findings of this study.

## Data Availability Statement

The original contributions presented in the study are included in the article/supplementary materials, further inquiries can be directed to the corresponding authors.

## Ethics Statement

The studies involving human participants were reviewed and approved by the Ethics Committee of Children's Hospital of Fudan University [(2017) 186]. Written informed consent to participate in this study was provided by the participants' legal guardian/next of kin. Written informed consent was obtained from the minor(s)' legal guardian/next of kin for the publication of any potentially identifiable images or data included in this article.

## Author Contributions

YF was responsible for study design, data interpretation, experiment, and drafting of the manuscript. WH was responsible for experiment and date analysis. YW, JL, YZ, ZT, and SM were contributed to samples collection, data acquisition, and analysis. YZ and YH supervised the procedure, involved in study design, and critically revision and edition of the manuscript. All authors contributed to manuscript revision, read, and approved the submitted version.

## Conflict of Interest

The authors declare that the research was conducted in the absence of any commercial or financial relationships that could be construed as a potential conflict of interest.

## Publisher's Note

All claims expressed in this article are solely those of the authors and do not necessarily represent those of their affiliated organizations, or those of the publisher, the editors and the reviewers. Any product that may be evaluated in this article, or claim that may be made by its manufacturer, is not guaranteed or endorsed by the publisher.
